# Breast Cancer by Age at Diagnosis in the Gharbiah, Egypt, Population-Based Registry Compared to the United States Surveillance, Epidemiology, and End Results Program, 2004–2008

**DOI:** 10.1155/2015/381574

**Published:** 2015-10-01

**Authors:** Jennifer A. Schlichting, Amr S. Soliman, Catherine Schairer, Joe B. Harford, Ahmed Hablas, Mohamed Ramadan, Ibrahim Seifeldin, Sofia D. Merajver

**Affiliations:** ^1^Department of Epidemiology, University of Michigan School of Public Health, Ann Arbor, MI 48109, USA; ^2^Department of Epidemiology, University of Nebraska Medical Center, Omaha, NE 68198, USA; ^3^Division of Cancer Epidemiology and Genetics, National Cancer Institute, National Institutes of Health, Department of Health and Human Services, Bethesda, MD 20892, USA; ^4^Center for Global Health, National Cancer Institute, National Institutes of Health, Department of Health and Human Services, Bethesda, MD 20892, USA; ^5^Tanta Cancer Center, Tanta, Gharbiah 31111, Egypt; ^6^Department of Internal Medicine, University of Michigan Medical School, Ann Arbor, MI 48109, USA

## Abstract

*Objective*. Although breast cancers (BCs) in young women often display more aggressive features, younger women are generally not screened for early detection. It is important to understand the characteristics of young onset breast cancer to increase awareness in this population. This analysis includes all ages, with emphasis placed on younger onset BC in Egypt as compared to the United States.* Methods*. BC cases in the Gharbiah cancer registry (GCR), Egypt, were compared to those in the Surveillance, Epidemiology, and End Results (SEER) database. This analysis included 3,819 cases from the GCR and 273,019 from SEER diagnosed 2004–2008.* Results*. GCR cases were diagnosed at later stages, with <5% diagnosed at Stage I and 12% diagnosed at Stage IV. 48% of all SEER cases were diagnosed at Stage I, dropping to 30% among those ≤40. Significant differences in age, tumor grade, hormone receptor status, histology, and stage exist between GCR and SEER BCs. After adjustment, GCR cases were nearly 45 times more likely to be diagnosed at stage III and 16 times more likely to be diagnosed at stage IV than SEER cases.* Conclusions*. Future research should examine ways to increase literacy about early detection and prompt therapy in young cases.

## 1. Introduction 

Breast cancer (BC) in young women in the United States (US) is a relatively rare occurrence [1]. However, BC is the leading cause of cancer death in adult women less than 60 years of age in high-income countries [2]. Furthermore, numerous studies have reported that BCs in young women tend to display more aggressive features, such as larger tumor size, poor differentiation, positive lymph nodes, high proliferation rates, higher incidence of Human Epidermal Growth Factor Receptor 2 (HER2/neu) overexpression, abnormal p53 expression, DNA aneuploidy, estrogen receptor/progesterone receptor negativity, and more tumors of the basal-like histologic subtype [3–10].

Studies dating back over thirty years have reported a high proportion of “rapidly progressing breast cancer” (RPBC) with inflammatory BC (IBC) characteristics in the North African country of Tunisia [11–15]. Findings from these Tunisian studies have spurred interest in further BC research in the mid-East, and particularly in North Africa. Recent BC studies in the region have focused on Egypt, aided by the founding of the Gharbiah population-based cancer registry (GCR) in 1998 as part of the Middle East Cancer Consortium (MECC) [16].

No studies have systematically examined younger onset BCs in a population-based cancer registry in a North African country to address the question of whether these observed differences are based on real differences in incidence and characteristics between regions of the globe. To this end, this study compared BC cases in the GCR to those in the US Surveillance, Epidemiology, and End Results (SEER) database, stratified by age. Between-country differences for younger and later onset BCs may indicate different etiologies and may have important implications for public health BC prevention efforts and clinical practice.

## 2. Material and Methods

### 2.1. Data Sources

Data sources for this study include the GCR in Tanta, Egypt, and the US SEER database. The GCR is based 90 Km north of Cairo in the Nile Delta region. The GCR is a population-based registry covering the Gharbiah Governorate of Egypt. The registry was established in 1998 within the US National Cancer Institute sponsored MECC joint cancer registration project, with case registration beginning in 1999. The GCR is jointly sponsored by MECC and the Egyptian Ministry of Health and Population based in Cairo and is housed in the Tanta Cancer Center. Medical doctors affiliated with the Tanta Cancer Center conduct active surveillance for all incident cancer cases among the approximately 3.4 million residents of Gharbiah diagnosed within and outside the governorate. Data are gathered through regular visits to all government, nongovernment, and private centers and laboratories where cancer patients are diagnosed and treated. Data collectors regularly visit and collect data from centers outside Gharbiah for residents of the governorate who are diagnosed or treated in other neighboring governorates or at the National Cancer Institute in Cairo. Data are also collected from the death certificates and the electronic mortality database of the governorate. While cancer case reporting is not required by law, a ministerial decree issued to request collaboration with the registry has aided data collection efforts [16]. The GCR data is subjected to routine quality control and validation and has been included in publications of the US National Cancer Institute (MECC reports) and the Cancer Incidence in Five Continents monograph of the International Agency for Research on Cancer [16–18].

The other data source for this paper was the SEER 18 Registries database. The dataset includes all BC cases from 2000 forward for the following SEER registries: San Francisco-Oakland, Connecticut, Detroit (metropolitan area), Hawaii, Iowa, New Mexico, Seattle (Puget Sound), Utah, Atlanta (metropolitan area), San Jose-Monterey, Los Angeles, Alaska Natives, rural Georgia, California excluding San Francisco/San Jose-Monterey/Los Angeles, Kentucky, Louisiana, New Jersey, and greater Georgia [19].

### 2.2. Data Management and Statistical Analysis

Variables of interest that were available in both datasets included age at diagnosis (continuous), tumor grade, International Classification of Diseases for Oncology-3 (ICD-O-3) histology [20], estrogen and/or progesterone receptor expression (positive versus negative), and American Joint Committee on Cancer (AJCC) stage group [21]. We also present frequencies and incidence rates by race and Hispanic ethnicity for the US SEER cases; race/ethnicity data was not available for GCR cases and therefore not included in multivariate analysis. ICD-O-3 histology codes examined included mucinous type (8480, 8481); duct type, not otherwise specified (NOS; 8500); medullary type (8510, 8512, 8513); lobular type, NOS (8520); duct and lobular type (8522); Paget disease, mammary type (8540, 8541, 8543); and sarcomas, including phyllodes tumor (8935–8982, 9020). Due to an extremely small number of cases in the GCR (each less than 1% of total cases), papillary type (8050, 8260, 8503), tubular type (8211), inflammatory type (8530), duct type mixed with other carcinoma types (8523), and lobular type mixed with other carcinoma types (8524) were grouped into an “other” category along with all other histologies [20]. AJCC 6th edition staging information was available in each dataset for the years 2004 to 2008. This analysis therefore includes all adult (18+ years of age) malignant female breast cancer cases diagnosed from 2004 to 2008 (excluding ductal and lobular in situ/Stage 0 cases and lymphomas).

GCR BC cases were compared to US SEER BC cases on each variable of interest initially through standard bivariate analysis. An independent group *t*-test was used to determine if there was a statistically significant difference between the mean ages of each population, while potential differences in all other variables, which were categorical, were tested using Pearson's chi-square statistic. A logistic regression model was fit to examine the multivariate association between country of residence and tumor characteristics, with the general model outlined below:

(1) $$\begin{array}{c} \mathrm{Logit}\left(p\right)=\beta_{0}+\beta_{1}\left(\text{age}\right)+\beta_{2}\left(\text{hormone receptor status}\right)+\beta_{3}\left(\text{grade}\right)+\beta_{4}\left(\text{histology}\right)+\beta_{5}\left(\text{stage}\right), \end{array}$$

where logit⁡(*p*) is the log odds of an Egyptian BC (versus one from the USA). For modeling purposes, hormone receptor status was considered positive if a case was estrogen and/or progesterone receptor positive and negative if a case tested negative for both of these hormone receptors or for estrogen receptor, if that was the only hormone receptor tested. Grade was categorized as low grade I/II versus high grade III/IV and histology as ductal, NOS versus other, and stage was grouped into four categories fit as indicator variables (I/referent, II, III, IV) based on AJCC stage group. All tests were two-tailed with an alpha level of 0.05.

In order to examine potential heterogeneity in tumor characteristics by age between the two countries and to account for age differences between the two populations, between-country differences were also examined within each of three age categories: ≤40 years of age (very young onset; likely premenopausal), 41–55 years of age (younger onset; pre/peri/postmenopausal), and ≥56 years (likely postmenopausal onset). In addition, age stratified incidence rates for each tumor characteristic were also calculated using yearly (2004–2008) population denominator estimates from the US and Egyptian census programs. Due to the structure of the Egyptian population denominator estimates, age categories were slightly modified to 25–39, 40–54, and ≥55 years of age for the purposes of incidence rate calculations. Incidence rates were calculated using SEER^*∗*^stat version 7.1.0 (National Cancer Institute, Bethesda, MD) and OpenEpi version 2.3.1 (Emory University, Atlanta, GA); all other analyses were conducted using SAS 9.3 (SAS Institute, Cary, NC).

## 3. Results

This analysis included 3,819 cases from the GCR and 273,019 from SEER. Four GCR cases were missing age at diagnosis and have been excluded from all analyses. No cases were missing histology. Grade information was missing for 1,029 (27%) GCR and 25,463 (9%) SEER cases. Complete hormone receptor information was missing for 1,886 GCR (49%) and 24,788 SEER (9%) cases, while 792 GCR (21%) and 18,221 SEER (7%) cases were missing complete stage information. However, 9 GCR and 609 SEER cases were able to be assigned to an overall stage III and were included in the logistic regression models. Cases missing information for a particular variable were excluded from analyses involving that variable.

Table 1 shows the GCR and SEER population characteristics for all ages combined and for cases ≤40 years of age, along with the *P* value derived from test statistics comparing the two populations on each variable. GCR and SEER cases differed significantly in age, grade, hormone receptor status, histology, and stage. The average age of GCR cases was over ten years younger than that of SEER cases (51.0 versus 61.4 years). The majority (88%) of GCR cases, including those ≤40 years of age, were diagnosed with grade II, or moderately differentiated tumors. However, the majority (56%) of younger (≤40 years of age) SEER cases were diagnosed with grade III, poorly differentiated tumors. Younger GCR and SEER cases also differed significantly on hormone receptor status, with 23% of GCR cases 40 years of age or younger being hormone receptor negative versus 33% of younger SEER cases. Histologies varied between the two populations, though, for both, the large majority were ductal, NOS. Among rarer histologies, for all ages combined and among younger cases, the GCR had a higher percentage of medullary type, mammary Paget disease, sarcomas, and “other,” while SEER had a higher percentage of mucinous type, lobular NOS, and mixed ductal and lobular type. The majority of SEER cases were White and non-Hispanic.

There was large, statistically significant variation in stage at diagnosis between the two populations. GCR cases were diagnosed at later stages, with less than 5% being diagnosed at Stage I and 12% being diagnosed at Stage IV. Forty-eight percent of SEER cases (all ages combined) were diagnosed at Stage I, although this dropped to 30% among those aged 40 and younger. Only 5-6% of SEER cases were diagnosed at Stage IV, even among those 40 years or younger. Cases aged 41–55 and those 56 and older followed a similar pattern to all ages combined with regard to differences in grade, hormone receptor status, histology, and stage (*P* < 0.01 for each; results not shown).

Table 2 shows the odds ratios (OR) and 95% confidence intervals (CI) obtained from age stratified logistic regression models examining the multivariate relationship between country of residence (Egypt's GCR versus US SEER) and BC characteristics. The first model combined all age groups and found younger age at diagnosis (OR = 0.95, 95% CI = 0.94–0.95), lower grade I/II (OR = 0.05, 95% CI = 0.04–0.06), negative hormone receptor status (OR = 1.95, 95% CI = 1.72–2.22), greater stage: Stage II (OR = 12.04, 95% CI = 9.69–14.97), Stage III (OR = 44.55, 95% CI = 35.84–55.38), Stage IV (OR = 16.04, 95% CI = 11.97–21.50) versus Stage I (referent), and duct, NOS histology (OR = 4.30, 95% CI = 3.5–5.07) were all independently associated with the GCR cases. The model examining cases ≤40 years of age generated similar results: OR for high versus low grade = 0.04, 95% CI = 0.02–0.06; negative versus positive hormone receptor status OR = 1.67, 95% CI = 1.26–2.22; Stage II OR = 10.57, 95% CI = 5.84–19.13; Stage III OR = 31.57, 95% CI = 17.43–57.19; Stage IV OR = 16.53, 95% CI = 8.12–33.64 versus Stage I referent, and duct, NOS versus all other histologies OR = 2.61, 95% CI = 1.82–3.75. Cases aged 41–55 and those over 56 years of age followed a similar pattern (results shown in Table 2).

Table 3 gives the country- and age-specific incidence rates per 100,000 woman-years and 95% CI for tumor grade, hormone receptor status, histology, AJCC stage, and race/Hispanic ethnicity for the US SEER cases. Incidence rates for the US SEER were generally higher with a few exceptions. Egyptian women < 40 years of age had a higher incidence of low grade I/II tumors (17.4 versus 11.9). Egyptian GCR cases had higher rates of Stages III and IV cancers across all age groups with the exception of stage IV cancers among women aged 55 and older. In the US SEER, White and non-Hispanic women had higher BC incidence rates with the exception of the 25–39-year-old age group, where the incidence rate for Black women was significantly higher.

## 4. Discussion

The results of this study reveal significant differences in age, tumor grade, hormone receptor status, histology, and stage between the BC cases included in Egypt's GCR and the US SEER registries. Egyptian GCR cases were, on average, over 10 years younger than US SEER cases, with nearly 19% of GCR cases ≤40 years of age as compared to only 6% of US SEER cases. Although there are age differences in the underlying population structure of the two countries [22, 23], we have accounted for these differences by conducting an age stratified analysis based on clinically and biologically relevant cut-points, as well as adjusting for age in the model that included all age groups combined. However, the possibility of residual confounding still exists. It should also be noted that Egypt's female life expectancy, while increasing greatly in the last 50 years from approximately 48 years in 1960 to 76 years for 2015, is still currently lower than the US 2015 estimate of 82 years [22].

As noted earlier, younger onset cases tend to have worse prognostic characteristics [3–10]. Furthermore, BC risk factors such as age at menarche, age at first full term birth (FFTB), parity, obesity, and oral contraceptive (OC) use have all been shown to demonstrate quantitative and qualitative age interactions with regard to BC risk [24, 25]. A review of 26 published manuscripts concluded that early age at menarche and later age at FFTB increase the risk of premenopausal more than postmenopausal BC risk [26], while Anderson et al. found age at menarche and FFTB interacted quantitatively, but not qualitatively, with age [24]. Nulliparity has been shown to decrease the risk of early onset BCs but increase the risk of BC in older women [24]. Body mass index (BMI) also qualitatively interacts with age with regard to BC risk. Higher BMI has been shown to have an inverse relationship with premenopausal BC risk, but a positive relationship with postmenopausal BC risk [25]. In a Polish case-control study, Anderson et al. also observed a qualitative interaction between OC use and age, where OC everuse was associated with decreased BC risk for women less than 40–44 years old, after which the risk was elevated for OC everusers [24].

Although we know average age at menopause is younger in Egyptian women as compared to the USA (46.7 versus 51 years in the USA) [27, 28], this likely does not explain the differences seen in cases ≤40 years of age. Nationally representative surveys in each country and recently published studies have shown that Egyptian women, on average, have a slightly older age at menarche, have more children and have them at a younger age, are less likely to use oral contraceptives, and have a slightly higher prevalence of obesity than US women [29–34]. Unfortunately, information on BC risk factors for individual cases was not available in either database for examination in this study, and race/ethnicity information is only available in the US SEER.

A high proportion of GCR cases, including those ≤40 years of age (88%), were assigned a tumor grade of II, moderately differentiated, as compared to SEER cases, where only 42% of all cases were grade II. This was also reflected in the incidence rates for tumor grade in this age group. However, among SEER cases ≤40 years of age, the majority (56%) were found to have grade III, poorly differentiated tumors, indicating these tumors may be more aggressive than those found in older women. It is unclear why there is such a large variation in grade between the two countries, although it should be noted that a large amount of GCR cases (27%) was missing grade information. The GCR and SEER programs use nearly identical rules for recording tumor grade, so it is unlikely that coding differences would be the sole explanation for grade differences between the registries [35–37]. However, it is possible that differences in pathology methods exist between the two countries that contribute to this large difference.

Grade II tumors have been shown to have the lowest concordance in reliability studies, as opposed to grades I and III tumors where miss-assignment of one to the other is rare. It is likely that many tumors on the border of being either grade I or III are upcoded or downcoded to grade II, thus increasing the proportion of grade II tumors. Furthermore, tissue sampling, handling, preservation, fixation, and preparation can all affect the accuracy and reliability of grade determination [38]. In addition, as neither registry program has a central laboratory for tissue examination, differences in accuracy, reliability, and general laboratory quality between both the GCR and the SEER registries as well as institutions contributing cases to each registry are to be expected. However, a recent study examining molecular subtypes of BC in archival tissue samples from the Universities of Cairo and Minya in Egypt also found over 80% of tumors were classified as grade II using Nottingham criteria, which might suggest better prognosis [39].

While a larger percentage of SEER cases ≤40 were hormone receptor negative as compared to GCR cases of the same age (33% versus 23%), in multivariate logistic regression models, the odds of GCR BC cases being hormone receptor negative were greater as compared to SEER cases across all age groups. Our results for estrogen and progesterone receptor status separately (not shown) are similar to those in the recent Egyptian case series by Salhia et al., where over all ages combined 65% of tumors were found to be estrogen receptor positive and 44% progesterone receptor positive [39]. Another study using GCR data from 2001 to 2006 also found the incidence of hormone receptor positive BC was higher than negative [40]. It should be noted that GCR variable recording, including hormone receptor status, is modelled after the SEER program, though unmeasured differences may exist between the two registry programs.

Interestingly, the percent of hormone receptor negative BC remained relatively flat across all age groups in the GCR data (approximately 22-23%), whereas in the US SEER data, the percent of hormone receptor negative cancers went from as high as 33% among women ≤40 years of age down to only 18% among women aged 56 years and older. It is unclear if this represents underlying etiologic or genetic differences between the two populations, or if this represents a type of cohort effect due to major changes in the lifestyle, reproductive, and environmental exposures that Egyptian women have experienced in the past few decades, namely, the modernization the county has undergone leading to an increase in exposure to the “western” lifestyle. This is especially true of adult weight-gain and postmenopausal obesity, which have been found to be associated with higher incidence of hormone receptor positive BC in multiple epidemiologic studies [41–43]. Recent estimates of obesity prevalence among Egyptian women are close to 40%, which is higher than recent estimates for US women [31, 34]. If these trends continue, it is possible that Egyptian postmenopausal BC rates may eventually equal those in the USA.

It should be noted that hormone receptor status testing is not necessarily routine practice in Egypt [39], and, in fact, only 51% of GCR cases had complete hormone receptor information available in this analysis, as opposed to 91% of US cases. However, due to an increase in hormone receptor testing in recent years in Egypt, this represents a higher proportion of cases with this information than in our previous study [40]. We conducted a sensitivity analysis in order to aid in determining what effect, if any, GCR cases missing hormone receptor status had on the results. GCR cases missing hormone receptor status were on average one year older, although there was no significant difference between those missing and not missing hormone receptor status by the three age categories. GCR cases missing hormone receptor status were also more likely to be diagnosed with duct and lobular type, mammary Paget disease, sarcomas, and “other” histologies, although as sarcomas are not amenable to hormonal treatment, it makes sense cases with these cancers may not necessarily be tested for hormone receptors. Those missing hormone receptor information were also more likely to be diagnosed with undifferentiated/anaplastic/Grade IV and Stage IV cancers, so it is possible women with more advanced cancers were not tested for hormone receptor status, if it was not expected to change management. However, when the models were rerun with those cases missing hormone receptor status (i.e., excluding hormone receptor status as a covariate), the results were similar to those presented in Table 2, indicating excluding those missing hormone receptor status did not have a strong effect on the overall model estimates.

In multivariate models, Egyptian GCR cases were more likely to be diagnosed with ductal, NOS BC as compared to the US SEER cases across all ages. Invasive ductal carcinoma is the most common type of BC, and prognosis is dependent on factors such as grade, hormone receptor status, and stage at diagnosis [44]. Among rarer histologies, the GCR had a higher percentage of medullary type, mammary Paget disease, sarcomas, and “other,” while SEER had a higher percentage of mucinous type, lobular NOS, and duct and lobular type. Due to small numbers of lobular cancers in the GCR, we were unable to examine these as a separate category in multivariate analysis.

One of the most striking results was that, after adjustment for age, grade, hormone receptor status, and histology, GCR cases were nearly 45 times more likely to be diagnosed at stage III and 16 times more likely to be diagnosed at stage IV than US SEER cases, with similar results across all age groups in age stratified analysis. Furthermore, this finding was also reflected in the country- and age stratified incidence rates by stage, where the incidence of stage III and IV cancers was higher in the Egyptian GCR regardless of age (with the exception of stage IV BC among women over 55 years of age). The possibility of an even higher proportion of unmeasured Stage IV cases in Egypt should also be noted if patients who perceive they have very advanced cancer choose not to seek treatment. Although it has been previously reported that Egyptian cases present with advanced disease [39], this is the first study to our knowledge to directly compare US to Egyptian BC cases on AJCC stage groups while adjusting for other established prognostic factors.

While it is possible that some unknown or unadjusted for tumor characteristic(s) exists that differs between these two populations and causes more aggressive disease in Egyptian women, and it should again be noted that a relatively large percentage of GCR cases were missing complete stage information (21%), a more likely explanation is that Egyptian women do not have their BC diagnosed as early in the disease process as US women, at all ages but especially at younger ages. If this is in fact the case, this finding represents a modifiable prognostic factor amenable to readily available educational and clinical interventions. Educating Egyptian women on the signs and symptoms of early onset BC and encouraging them to perform breast self-exams represent a relatively low cost and likely feasible strategy to intervene earlier in the disease process, when treatments are most efficacious. Increasing the availability of regular mammography screening to Egyptian women where resources are available may also lead to BC diagnosis at earlier stages in this population [39] but would not be necessarily useful in women <40, a population that is not screened in the USA either. In spite of not undergoing mammographic screening, women diagnosed <40 in the USA do not have a high incidence of stage III or IV breast cancer, supporting that literacy in the population about signs and symptoms leads to earlier diagnosis. Similarly, acculturated strategies could be tested and implemented in Egypt.

It has been suggested previously that mammography should be performed earlier in Arab populations due to the younger average age at onset [45]. However, as has been noted, this is driven largely by the younger age distribution of Arab populations and low and middle income countries in general [46, 47]. As has been seen in the USA, screening would likely increase the average age at diagnosis, with the US older average age at diagnosis likely being reflective of screening programs for women over 50 years of age. As seen in our analysis, when the age-specific incidence rates are compared, US women have a higher rate at all ages, though the difference is less pronounced in those <40 years of age.

In conclusion, Egyptian GCR and US SEER cases significantly differ in age at diagnosis, tumor grade, hormone receptor status, histology, and stage, with these differences in tumor characteristics persisting in age stratified and multivariate analysis. As detailed BC risk factor information was not available in either registry, it is unclear what factors are leading to the differences in age at diagnosis, tumor grade, hormone receptor status, and histology. There has been a recent and relatively large increase in the prevalence of obesity in Egypt, which represents a modifiable BC risk factor and area for future research in Egypt and other middle-income countries that now grapple with an increasing chronic disease burden traditionally associated with “western” lifestyle factors [48]. Most important for survival, GCR cases were much more likely to be diagnosed at later stages, and this prognostic factor is amenable to currently existing educational interventions and screenings. Registry improvements over time, especially for the relatively new GCR, will increase the completeness of case and variable recording, and multiple imputation may be considered for variables where incomplete recording persists. Future research should examine currently recognized as well as novel genetic and environmental factors that may contribute to the tumor characteristic differences between these two populations, including racial/ethnic diversity, as well as examining how these differences may contribute to treatment and survival differences.

## Figures and Tables

**Table 1:** Characteristics of Gharbiah, Egypt, and US SEER breast cancer cases, 2004–2008.

	Gharbiah Cancer Registry *n* and (%)		US SEER Registries *n* and (%)		*P* value	
Mean age at diagnosis (SD)	51.0 (11.5)		61.4 (14.3)		<0.0001^a^	

Age in years						
≤40	713 (18.7)		17,199 (6.3)			
41–54	1,842 (48.2)		84,122 (30.8)		<0.0001^b^	
≥55	1,264 (33.1)		171,698 (62.9)			

	All ages combined	≤40 years of age	All ages combined	≤40 years of age	All ages combined	≤40 years of age

Grade						
I	29 (1.0)	5 (0.9)	53,146 (21.5)	1,362 (8.6)	<0.0001^b^	<0.0001^b^
II	2,465 (88.4)	472 (88.2)	104,147 (42.1)	5,252 (33.0)		
III	241 (8.6)	49 (9.2)	86,815 (35.1)	8,945 (56.2)		
IV	55 (2.0)	9 (1.7)	3,448 (1.4)	369 (2.3)		
Missing (% of total cases)	1,029 (26.9)	178 (25.0)	25,463 (9.3)	1,271 (7.3)		

Hormone receptor						
Positive	1,495 (77.3)	278 (77.4)	197,482 (79.6)	10,583 (66.9)	0.02^b^	<0.0001^b^
Negative	438 (22.7)	81 (22.6)	50,749 (20.4)	5,233 (33.1)		
Missing (% of total cases)	1,886 (49.4)	354 (49.6)	24,788 (9.1)	1,383 (8.0)		

Histology^c^						
Mucinous	36 (0.9)	6 (0.8)	6,430 (2.4)	259 (1.5)	<0.0001^b^	<0.0001^b^
Duct, NOS	2,812 (73.6)	530 (74.3)	191,138 (70.0)	13,652 (79.4)		
Medullary	56 (1.5)	17 (2.4)	1,283 (0.5)	196 (1.1)		
Lobular	155 (4.1)	14 (2.0)	22,155 (8.1)	495 (2.9)		
Duct and lobular	57 (1.5)	22 (3.1)	19,992 (7.3)	892 (5.2)		
Paget disease, mammary	43 (1.1)	14 (2.0)	1,003 (0.4)	99 (0.6)		
Sarcomas; phyllodes tumor	46 (1.2)	14 (2.0)	703 (0.3)	127 (0.7)		
Other	614 (16.1)	96 (13.5)	30,315 (11.1)	1,479 (8.6)		

AJCC stage group						
I	144 (4.7)	22 (3.9)	122,936 (48.1)	4,887 (30.1)	<0.0001^b^	<0.0001^b^
IIA	632 (20.8)	108 (19.4)	60,856 (23.8)	4,643 (28.6)		
IIB	546 (18.0)	113 (20.3)	25,909 (10.1)	2,627 (16.2)		
IIIA	701 (23.1)	135 (24.2)	17,205 (6.7)	1,910 (11.8)		
IIIB	188 (6.2)	29 (5.2)	6,190 (2.4)	436 (2.7)		
IIIC	444 (14.6)	87 (15.6)	7,914 (3.1)	777 (4.8)		
IV	372 (12.3)	64 (11.5)	13,788 (5.4)	937 (5.8)		
Missing (% of total cases)	792 (20.7)	155 (21.7)	18,221 (6.7)	982 (5.7)		

Race						
White	—	—	223,957 (82.0)	12,609 (73.3)	—	—
Black	—	—	27,630 (10.1)	2,580 (15.0)		
American Indian/Alaska Native	—	—	1,256 (0.5)	108 (0.6)		
Asian/Pacific Islander	—	—	18,702 (6.9)	1,761 (10.2)		
Other/unknown	—	—	1,474 (0.5)	141 (0.8)		

Hispanic	—	—	25,017 (9.2)	3,024 (17.6)	—	—
Non-Hispanic	—	—	248,092 (90.8)	14,175 (82.4)		

^a^Based on *t*-test comparing Egyptian and US breast cancer cases, missing not included.

^b^Based on chi-square test comparing Egyptian and US breast cancer cases, missing not included.

^c^No cases missing histology; —: race/ethnicity unavailable in GCR.

**Table 2:** Odds ratios and 95% confidence intervals from age stratified logistic regression models examining the relationship between country of residence and breast cancer characteristics, Egypt's GCR, and US SEER cases, 2004–2008^a^.

Variable	Odds ratio (95% confidence interval)			
	Model 1: all ages combined	Model 2: ≤40 Years of age	Model 3: 41–55 Years of age	Model 4: ≥56 Years of age
Age (continuous)	0.95 (0.94–0.95)	—	—	—

Grade				
Low (I/II)^b^	1.00	1.00	1.00	1.00
High (III/IV)	0.05 (0.04–0.06)	0.04 (0.02–0.06)	0.05 (0.04–0.06)	0.07 (0.05–0.10)

Hormone Receptor				
Positive^b^	1.00	1.00	1.00	1.00
Negative	1.95 (1.72–2.22)	1.67 (1.26–2.22)	2.19 (1.83–2.62)	1.87 (1.50–2.33)

Histology				
Other^b^	1.00	1.00	1.00	1.00
Duct, NOS	4.30 (3.65–5.07)	2.61 (1.82–3.75)	4.62 (3.64–5.88)	5.37 (4.02–7.19)

AJCC stage Group				
I^b^	1.00	1.00	1.00	1.00
II	12.04 (9.69–14.97)	10.57 (5.84–19.13)	10.99 (8.14–14.85)	14.93 (10.29–21.65)
III	44.55 (35.84–55.38)	31.57 (17.43–57.19)	40.96 (30.35–55.28)	64.68 (44.65–93.70)
IV	16.04 (11.97–21.50)	16.53 (8.12–33.64)	11.87 (7.56–18.64)	21.23 (13.16–34.28)

^a^All variables in each column are mutually adjusted for each other.

^b^Referent Category.

**Table 3:** Age stratified average incidence rates by country of residence and breast cancer characteristics, Egypt's GCR, and US SEER cases, 2004–2008.

Variable	Incidence rate per 100,000 woman-years (95% confidence interval)					
	25–39 years of age		40–54 years of age		≥55 years of age	
Registry	Egypt's GCR	US SEER	Egypt's GCR	US SEER	Egypt's GCR	US SEER

Grade						
Low (I/II)	17.4 (15.7–19.2)	11.9 (11.6–12.3)	76.5 (72.3–80.9)	95.2 (94.3–96.1)	91.2 (85.3–97.4)	221.4 (220.1–222.7)
High (III/IV)	2.3 (1.7–3.0)	17.9 (17.5–18.3)	8.2 (6.9–9.7)	68.5 (67.7–69.3)	11.8 (9.7–14.1)	104.7 (103.8–105.6)

Hormone Receptor						
Positive	10.3 (9.0–11.7)	19.4 (19.0–19.8)	45.2 (42.0–48.5)	125.9 (124.9–127.0)	56.4 (51.8–61.3)	268.3 (266.8–269.7)
Negative	2.8 (2.1–3.5)	10.2 (9.9–10.5)	13.8 (12.1–15.7)	38.4 (37.9–39.0)	16.0 (13.6–18.7)	58.9 (58.2–59.6)

Histology						
Other	6.3 (5.3–7.4)	6.6 (6.3–6.8)	29.8 (27.2–32.6)	47.6 (47.0–48.3)	40.5 (36.6–44.7)	117.7 (116.7–118.6)
Duct, NOS	19.5 (17.7–21.4)	25.6 (25.1–26.1)	84.1 (79.8–88.7)	130.0 (128.9–131.0)	107.0 (100.6–113.7)	245.9 (244.5–247.3)

AJCC stage group						
I	0.9 (0.6–1.4)	8.6 (8.4–8.9)	4.6 (3.6–5.7)	73.0 (72.2–73.8)	5.3 (3.9–6.9)	174.9 (173.7–176.1)
II	7.5 (6.4–8.7)	13.8 (13.4–14.1)	37.7 (34.8–40.8)	63.6 (62.8–64.3)	42.2 (38.2–46.5)	105.4 (104.5–106.3)
III	9.8 (8.6–11.2)	6.1 (5.9–6.3)	40.3 (37.3–43.5)	24.4 (24.0–24.9)	49.6 (45.2–54.2)	36.9 (36.4–37.5)
IV	2.4 (1.8–3.1)	1.8 (1.7–2.0)	9.2 (7.8–10.8)	7.5 (7.2–7.7)	18.0 (15.4–20.8)	19.5 (19.1–19.9)

Race^a^						
White	—	30.0 (29.4–30.6)	—	171.3 (169.9–172.7)	—	350.1 (348.3–351.9)
Black	—	34.6 (33.1–36.2)	—	163.9 (160.6–167.3)	—	312.3 (307.4–317.4)
AI/AN	—	14.8 (11.9–18.3)	—	81.6 (74.3–89.5)	—	155.9 (144.1–168.4)
API	—	27.5 (26.1–29.0)	—	152.0 (148.4–155.7)	—	232.6 (227.9–237.3)

Hispanic	—	23.5 (22.6–24.5)	—	125.8 (123.2–128.4)	—	238.9 (234.7–243.2)
Non-Hispanic	—	32.5 (31.8–33.1)	—	176.6 (175.2–177.9)	—	347.8 (346.0–349.5)

Total	25.7 (23.7–27.9)	32.2 (31.7–32.7)	113.9 (108.8–119.2)	177.6 (176.4–178.8)	147.5 (139.9–155.4)	363.5 (361.8–365.2)

^a^AI/AN: American Indian/Alaska Native; API: Asian/Pacific Islander; SEER^*∗*^stat cannot calculate other/unknown category incidence rates; —: race/ethnicity unavailable in GCR.
